# Global, regional, and national burden of gastritis and duodenitis from 1990 to 2021 with projections to 2050: a systematic analysis of the Global Burden of Disease Study 2021

**DOI:** 10.7150/ijms.109762

**Published:** 2025-05-10

**Authors:** Lufei Wang, Wei Jiang, Hui Li

**Affiliations:** 1 State Key Laboratory of Genetic Engineering, School of Life Sciences, Fudan University, Shanghai 200438, China; 2 Ministry of Education Key Laboratory of Contemporary Anthropology, School of Life Sciences, Fudan University, Shanghai 200438, China; 3 Human Phenome Institute, Zhangjiang Fudan International Innovation Center, Fudan University, Shanghai 200438, China

**Keywords:** Global Burden of Diseases, Gastritis and duodenitis, Socio-demographic index (SDI), Prevalence, Disability-adjusted life-years (DALYs)

## Abstract

**Background:** Gastritis and duodenitis are highly prevalent upper gastrointestinal inflammatory conditions. We estimate the most up-to-date global, regional, and national burden in incidence, prevalence, mortality, and disability-adjusted life-years (DALYs) for gastritis and duodenitis from 1990 to 2021 with projections to 2050.

**Methods:** Population-based data in this study was retrieved from the Global Burden of Diseases, Injuries, and Risk Factors Study 2021 (GBD 2021). We evaluated the temporal trends of age-standardized rates of gastritis and duodenitis prevalence (ASPR), incidence (ASIR), mortality (ASMR), and DALYs (ASDR) across 204 countries and territories, as well as estimated annual percentage changes (EAPC) from 1990 to 2021. Analyses were stratified by sex, age subgroup, socio-demographic index (SDI) at the global, regional, and national level. A Bayesian age-period-cohort model was employed to project ASPR and ASDR of gastritis and duodenitis by sex and age up to 2050.

**Results:** In 2021, 27.20 million (95% UI: 21.85-33.65) individuals globally had gastritis and duodenitis, with an ASIR of 323.24 (95% UI: 261.35-398.64) per 100,000. Globally, the prevalent cases of gastritis and duodenitis in 2021 was higher in females than in males and increased with age, peaking at the fifth decade of life. The number of prevalent cases of gastritis and duodenitis is projected to reach approximately 51.24 million by 2050. In 2021, gastritis and duodenitis caused 2.81 (95% UI: 2.17-3.61) million DALYs worldwide, with an ASDR of 35.64 (95% UI: 27.56-45.77) per 100,000, while the ASMR was 0.54 (95% UI: 0.47-0.62) per 100,000. Despite global decreases in ASPR and ASIR, increasing trends were observed in the low and low-middle SDI regions from 1990 to 2021. The low SDI regions exhibited the highest ASDR of 63.44 (95% UI: 44.08-87.16) per 100,000. Among 21 GBD regions, East Asia exhibited the highest ASPR and ASIR in 2021. The ASPR of gastritis and duodenitis in 2050 is forecast to be 402.14 per 100,000, with an ASDR of approximately 22.09 per 100,000.

**Conclusion:** Despite global reductions in ASPR, ASIR, ASMR, and ASDR between 1990 and 2021, the global burden of gastritis and duodenitis exhibits regional disparities, closely linked to SDI levels. The highest burden of gastritis and duodenitis was observed in East Asia and Sub-Saharan Africa. More potent measures are urgently needed in low SDI regions to forestall the increase of Gastritis and duodenitis burden.

## Introduction

Gastritis and duodenitis are heterogeneous chronic inflammatory conditions of the upper gastrointestinal tract 1-3. Long-term gastritis and duodenitis can lead to severe symptoms, such as abdominal pain, nausea, bloating, and early satiety, further elevating the risk of gastric cancer 4. *Helicobacter pylori* (*H. pylori*) infection is a critical risk factor of gastritis and duodenitis, linked to the development of precancerous lesions that can cause gastric cancer 5, 6. Given the high mortality and poor prognosis associated with gastric cancer 7, particular attention should be paid to the prevalent trends and disease burden of gastritis and duodenitis, particularly chronic atrophic gastritis 8 and intestinal metaplasia 9.

Previous studies have reported the global trends in gastritis and duodenitis, including their incidence, prevalence, mortality, and disability-adjusted life-years (DALYs), as well as the analysis of risk factors 10, 11. The recent study indicates a rising prevalence and incidence of gastritis and duodenitis, despite a decrease in age-standardized rates 11, highlighting a substantial ongoing global burden. However, these studies have not offered detailed analyses by sex, age subgroup, socio-demographic index (SDI), or projection of global trends. Additionally, despite the increasing screening efforts of *H. pylori*, the annual reinfection rate after eradication remains higher (5%-10%) among adults in developing countries 5. This suggests an unstable prevalence of gastritis and duodenitis, warranting further investigation. With the release of the Global Burden of Diseases, Injuries, and Risk Factors Study 2021 (GBD 2021) 12, a comprehensive assessment is crucial to understand the complex patterns of global gastritis and duodenitis and their associated disease burden.

Here, we present an updated assessment of the temporal trends of age-standardized rates of gastritis and duodenitis prevalence (ASPR), incidence (ASIR), mortality (ASMR), and DALYs (ASDR), as well as estimated annual percentage changes (EAPC) from 1990 to 2021. We conducted a descriptive stratification analysis by sex, age, and SDI across different countries and territories. Furthermore, we examined the correlations between SDI and age-standardized rates to evaluate the predictive power of SDI as a forecasting index for the global burden of gastritis and duodenitis. Finally, we utilized a Bayesian age-period-cohort (BAPC) model to project the global trends of gastritis and duodenitis up to 2050. Through the most up-to-date and comprehensive assessment, we aim to provide essential information for policy makers and researchers to develop active surveillance strategies for prevention and treatment of gastritis and duodenitis.

## Materials and Methods

### Data acquisition

The data in this study were sourced from the Global Burden of Diseases, Injuries, and Risk Factors Study (GBD) Collaborator Network, in accordance with the GBD Protocol. The GBD 2021 was established via a systematic review of the literature from the published report, databases, and search engines, covering 371 diseases and injuries with 88 risk factors across 204 countries and territories from 1990 to 2021 12, 13. DisMod-MR 2.1, a disease-model-Bayesian meta-regression model, was employed by the GBD 2021 to compute incidence and prevalence. According to the GBD 2021 metrics, all calculations were conducted 500 times to generate draw-level estimates. Final estimates represent the mean estimate across 500 draws, and 95% uncertainty interval (UI) are represented by the 2.5th and 97.5th percentile values across the draws 12. Our analysis estimated gastritis and duodenitis prevalence, incidence, mortality, and disability-adjusted life-years (DALYs), stratified by age and sex across 204 countries and territories from 1990 to 2020. This study followed the Guidelines for Accurate and Transparent Health Estimates Reporting (GATHER) 14.

Although gastritis and duodenitis refer to inflammation of the mucosal lining of the stomach and duodenum, respectively, the GBD 2021 adopts the common practice of using these terms to describe gastropathy and duodenopathy, meaning any form of injury to the mucosal lining of the stomach and duodenum, be it inflammatory (such as due to infection or autoimmune disease) or otherwise (such as due to nonsteroidal anti-inflammatory medications), regardless of symptoms (https://www.healthdata.org/gbd/methods-appendices-2021/gastritis-and-duodenitis-0). In practice and in the GBD, both inflammatory and non-inflammatory mucosal damage are classified together as gastritis and duodenitis. This can result from diverse aetiologies but is primarily due to infection with *H. pylori* or the abuse of non-steroidal anti-inflammatory drugs. The GBD 2021 used diagnosis codes from the International Classification of Diseases (ICD, version 10) for gastritis and duodenitis (ICD-10 code: K29).

### Socio-demographic index (SDI)

The socio-demographic index (SDI) is an index of the overall development level of a country or region based on the total fertility rate (< 25 years), mean education level for those aged 15 years and older, and lag-distributed income per capita 12, 15. This study classified 204 countries and territories into low, low-middle, middle, high-middle, and high SDI regions based on their SDI levels. The SDI scale ranges from 0 (worst) to 1 (best), with higher values indicating a higher development level. SDI was used to determine the relationship of a country or territory's socioeconomic development status with ASPR, ASIR, ASMR, and ASDR.

### Statistical analysis

We evaluated the temporal trends of ASPR, ASIR, ASMR, ASDR, and computed annual percentage changes (EAPC). The age-standardized rate (ASR) per 100,000 individuals was computed using the following formula:



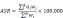



where 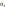
and 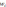
represent the age-specific rate and the number of people in the corresponding age group among the world standard population 12. *A* and *i* represent the number of age groups and the age class.

The EAPC was used to estimate the change of ASPR, ASIR, ASMR, and ASDR during different periods. The EAPC was computed using a linear regression model: Y = α+βX+e, where Y is the natural logarithm of the ASR, X is the year, and α and β are the intercept and slope. The 95% confidence interval (CI) for the EAPC estimate is given by the range between the lower confidence limit and the upper confidence limit. A positive lower confidence limit of the EAPC 95% CI indicates an increasing trend in the corresponding ASR, while a negative upper confidence limit suggests a decreasing trend. If the EAPC 95% CI includes 0, the ASR is considered stable. We combined a Bayesian age-period-cohort (BAPC) model with Integrated Nested Laplace Approximations (INLA) to forecast global prevalence trends of gastritis and duodenitis up to 2050. This projection utilized the BAPC and INLA packages 16, 17, which could accurately and reliably forecast trends according to previous studies 18, 19. The statistical analysis and figure generation were performed using the R software (version 4.4.0).

## Results

### Global level

**Figure 1:**
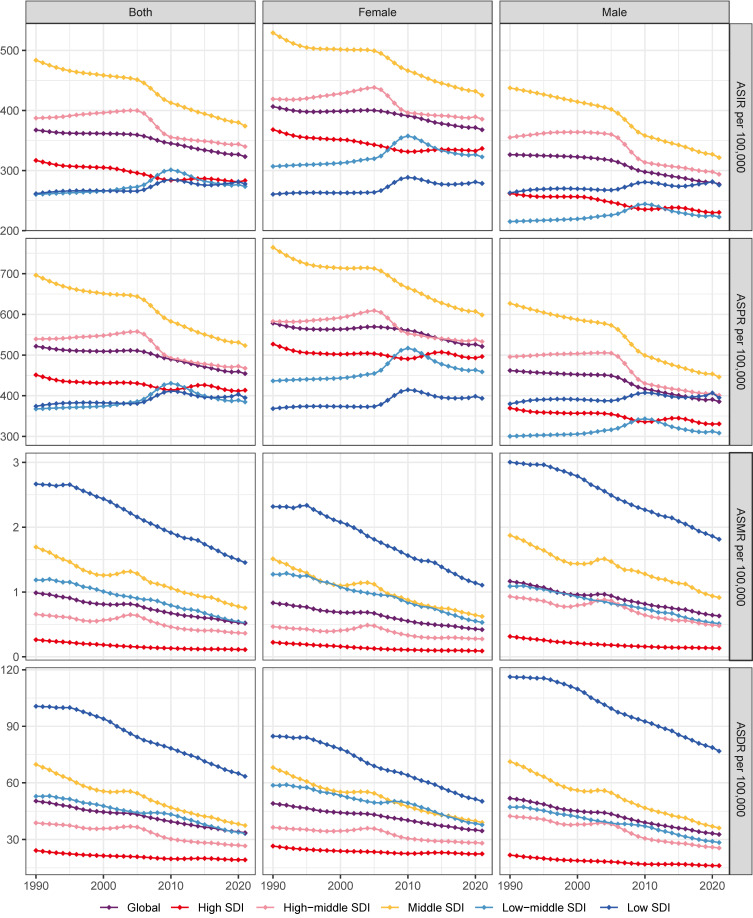
The temporal trends of the ASPR, ASIR, ASMR, ASDR due to gastritis and duodenitis by sex and SDI regions from 1990 to 2021.

**Figure 2:**
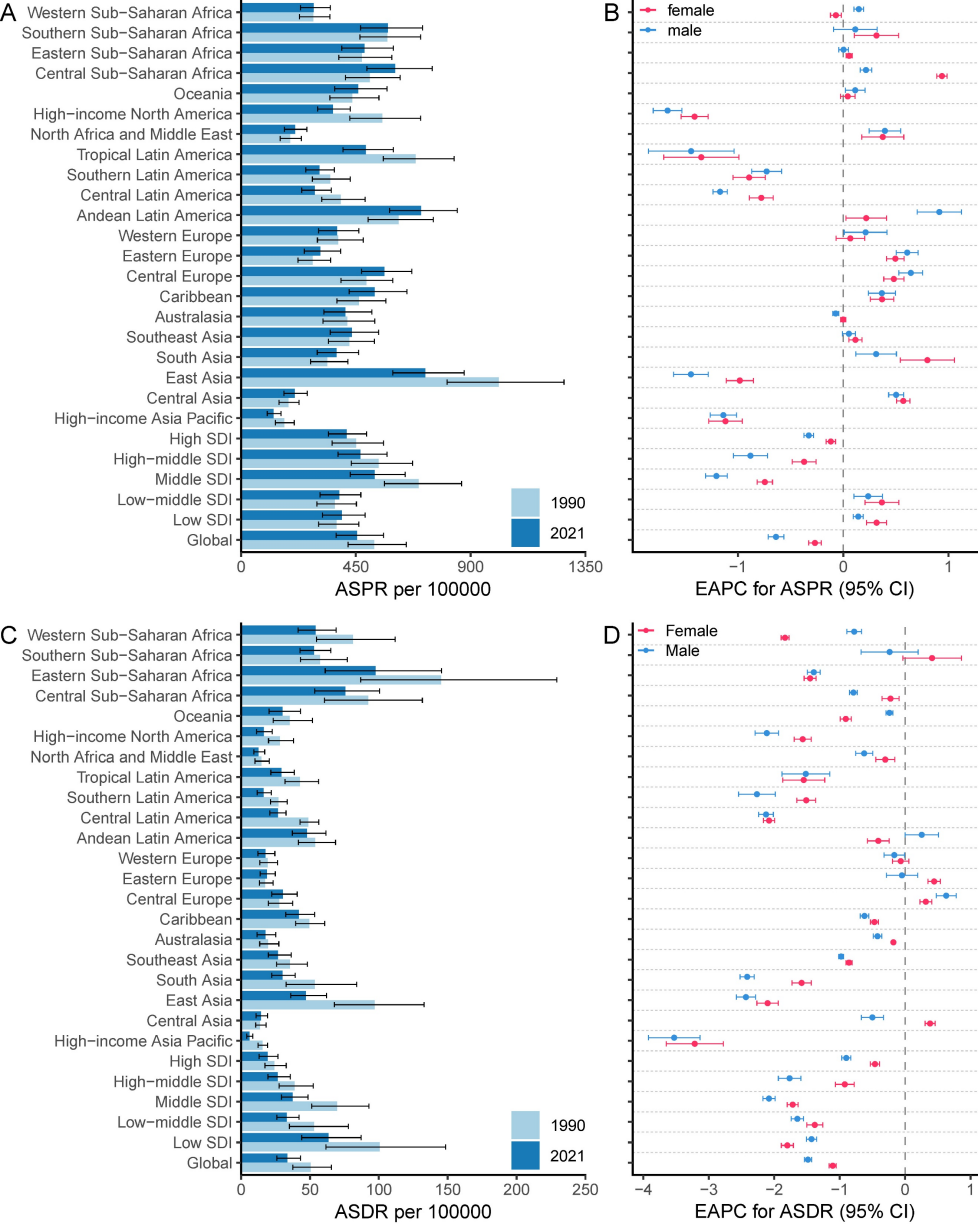
The ASPR and ASDR due to gastritis and duodenitis between 1990 and 2021, and the corresponding EAPC by sex from 1990 to 2021.

In 2021, the global incident cases of gastritis and duodenitis reached 27,202,524 (95% UI: 21,851,453-33,647,647), representing an increase of 57.12% since 1990. However, the ASIR of duodenitis decreased by 12.05%, from 367.51 (95% UI: 295.09-459.29) per 100,000 in 1990 to 323.24 (95% UI: 261.35-398.64) per 100,000 in 2021 (Figure 1, Figure S1, Table S1). In 2021, there were 38,293,409 (95% UI: 31,167,468-47,226,499) individuals with gastritis and duodenitis globally, which represents an increase of 56.71% from 1990. The ASPR in gastritis and duodenitis decreased by 12.91%, from 521.67 (95% UI: 419.28-647.52) per 100,000 in 1990 to 454.31 (95% UI: 372.66-558.27) per 100,000 in 2021, with an EAPC of -0.43 (95% CI: -0.49 to -0.37) (Figure 1, Figure 2A-B, Table S1). Between 1990 and 2021, the ASMR and ASDR decreased by 47.47% and 33.38%, with corresponding EAPC of -2.03 (95%CI: -2.15 to -1.92) and -1.3 (95%CI: -1.35 to -1.24), respectively (Figure S1, Figure 2C-D). However, the total number of prevalent cases increased by 56.6%, from 24,436,602 (95% UI: 19,731,576-30,046,057) to 38,293,409 (95% UI: 31167469-47226499); the number of deaths increased by 13.16%, from 38,038 (95% UI: 26,421-52,809) to 43,301 (95% UI: 35,853-51,140) and total DALYs increased by 62.33%, from 2,302,039 (95% UI: 1,709,839-3,002,698) to 2,775,629 (95% UI: 2,148,698-3,550,077).

### Regional and national level

The global burden of gastritis and duodenitis varies across regions and is associated with socio-demographic index (SDI). The ASPR in the low and low-middle SDI regions has shown an increasing trend from 1990 to 2021, with corresponding EAPC of 0.24 (95%CI: 0.17 to 0.3) and 0.33 (95%CI: 0.18 to 0.48), respectively. In contrast, the ASPR in the high, high-middle, and middle SDI regions has shown a decreasing trend, with the corresponding EAPC of -0.23 (95%CI: -0.27 to -0.19), -0.6 (95%CI: -0.73 to -0.46), and -0.95 (95%CI: -1.03 to -0.86), respectively (Figure 2A-B, Table S1). Among these regions, the middle SDI regions have shown the most substantial decline in ASPR, with the ASPR of 523.25 (95% UI: 428.09- 643.39) per 100,000 in 2021. Conversely, the low-middle SDI regions have shown the lowest ASPR of 384.61 (95% UI: 309.45-469.52) per 100000 (Figure 2A, Table S1). These regional disparities based on SDI levels reveals a complex interplay of multiple factors, such as improving diagnostic capabilities, increasing life expectancy, and increasing exposure to risk factors. The ASIR also demonstrated similar regional disparities (Figure 1, Figure S1). Additionally, the ASMR and ASDR further highlighted distinct patterns among different SDI regions. Overall, the ASDR among different SDI levels decreased from 1990 to 2021, of which middle SDI regions showed the highest decline, with an EAPC of -1.92 (95% CI: -2 to -1.83) (Figure 1, Figure 2D, Table S1). In 2021, the low SDI regions bore the highest burden of gastritis and duodenitis, with an ASDR of 63.44 (95% UI: 44.08-87.16) per 100,000, while high SDI regions exhibited the lowest ASDR of 19.24 (95% UI: 13.15-26.71) per 100,000 (Figure 1, Figure 2C, Table S1). These results suggest a complex relationship between SDI levels and various prevalence metrics at different stages of epidemiological transition, necessitating further analysis across different geographical regions.

Among 21 GBD regions, the highest ASPR of gastritis and duodenitis in 2021 was in East Asia (721.66 [95%UI: 594.36-875.06] per 100,000), Andean Latin America (705.21 [95%UI: 582.53-847.45] per 100,000), Southern Sub-Saharan Africa (603.8 [95%UI: 493.07-749.34] per 100,000) in 2021 (Figure 2A-B, Table S1). In contrast, the lowest ASPR was in High-income Asia Pacific (127.33 [102.67- 156.54] per 100,000), Central Asia (210.60 [168.14- 259.02] per 100,000), and North Africa and Middle East (211.75 [169.70- 257.89] per 100,000). The highest ASPR was 5.67 times higher than the lowest ASPR. In 2021, the highest ASDR and ASMR were observed in Eastern Sub-Saharan Africa (97.53 [95% UI: 61.07-145.49] per 100,000; 2.45 [95% UI: 1.38-4.01] per 100,000), Central Sub-Saharan Africa (75.76 [95% UI: 53.51-100.54] per 100,000; 1.99 [95% UI: 1.18-2.9] per 100,000), Western Sub-Saharan Africa (54.05 [95% UI: 41.4-68.99] per 100,000; 1.32 [95% UI: 1.01-1.68] per 100,000), and Southern Sub-Saharan Africa (52.93 [95% UI: 42.79-65.19] per 100,000; 1.29 [95% UI: 1.12-1.47] per 100,000), East Asia (47.09 [95% UI: 36.02-62.08] per 100,000; 0.97 [95% UI: 0.78-1.19] per 100,000). Conversely, High-income Asia Pacific had the lowest ASDR (0.03 [95% UI: 0.02-0.05] per 100,000) and ASMR (6.04 [95% UI: 4.02-8.48] per 100,000) (Figure 2C-D, Figure S1, Table S1). Due to East Asia's high ASIR and large population, this region accounted for over a third of new cases of gastritis and duodenitis globally in 2021 (10016836.79 [95% UI: 7935715.89-12479446.19] of 27202523.83 [95% UI: 21851452.89-33647646.83] global incidence number). East Asia and Andean Latin America exhibited the highest ASPR and ASIR, whereas Sub-Saharan Africa exhibited the highest ASMR and ASDR. On the other hand, the High-income Asia Pacific not only showed the lowest ASPR and ASIR but also the lowest ASDR and ASMR, which may be associated with its high SDI levels. Overall, GBD regions with higher ASIR and ASPR tended to have higher ASMR and ASDR with minor changes in rank order.

**Figure 3:**
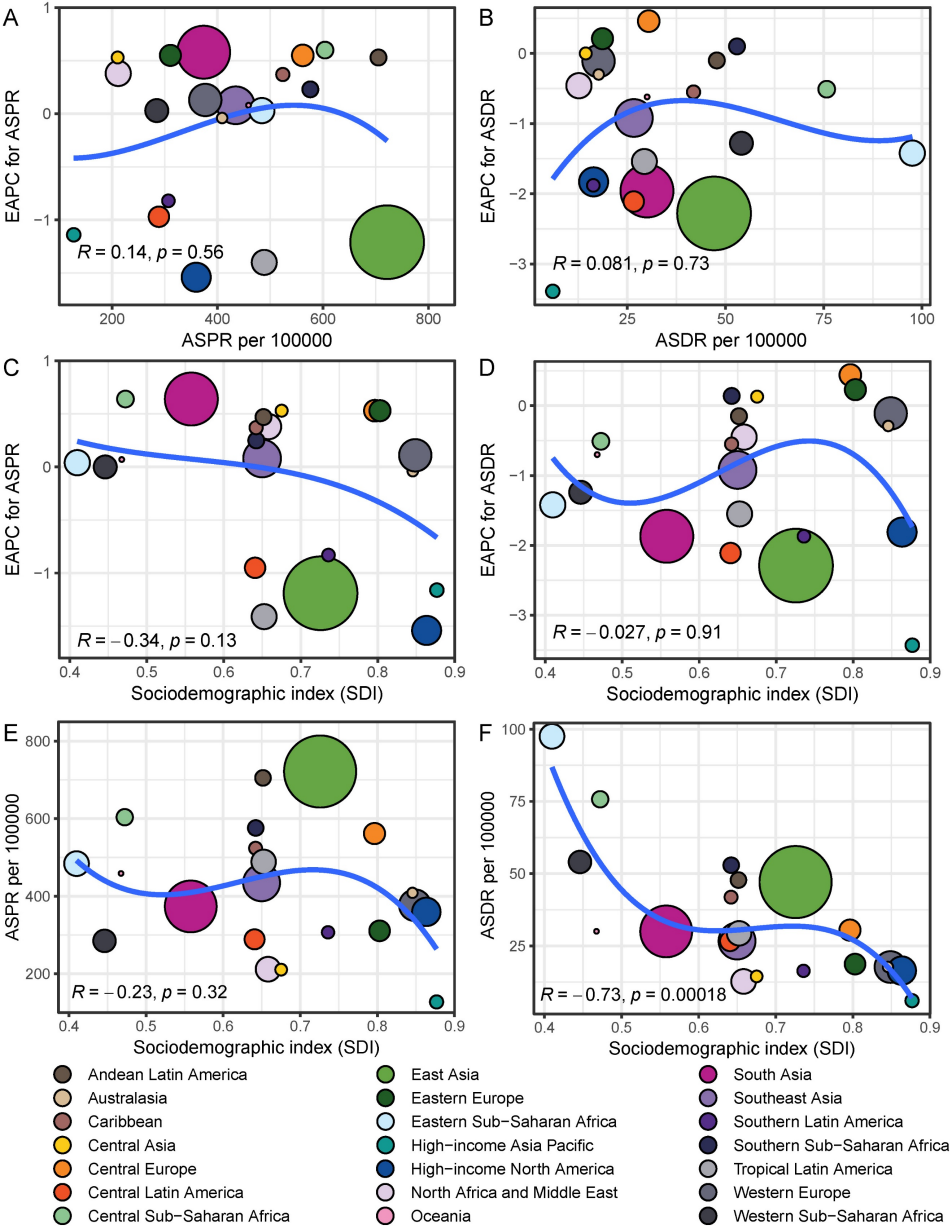
The relationships of EAPC and SDI with ASPR and ASDR among different regions in 2021. The size of points represents the corresponding number of prevalent cases and DALYs.

Among 21 GBD regions, the regional ASPR for gastritis and duodenitis decreased in High-income North America, Tropical Latin America, East Asia, High-income Asia Pacific, Central Latin America, and Southern Latin America between 1990 and 2021, with the corresponding EAPC of -1.54 (95% CI: -1.67 to -1.4), -1.41 (95% CI: -1.78 to -1.03), -1.19 (95% CI: -1.34 to -1.05), -1.16 (95% CI: -1.3 to -1.01), -0.95 (95% CI: -1.04 to -0.87), -0.83 (95% CI: -0.97 to -0.68), respectively (Figure 2B, Figure 2D, Table S1). Similar regional trends to ASPR were seen in the ASIR of gastritis and duodenitis from 1990 to 2021 (Figure 2B, Figure S1, Table S1). Between 1990 and 2021, the ASDR of gastritis and duodenitis decreased in all GBD regions except for Eastern Europe (0.23 [95% CI: 0.10 to 0.36]), Central Europe (0.44 [95% CI: 0.33 to 0.55]), and Central Asia (0.13 [95% CI: 0.05 to 0.21]) (Table S1). Of these three regions, the ASDR of Central Europe showed an upward trend among both females and males, whereas the ASDR of Eastern Europe and Central Asia increased exclusively among females between 1990 and 2021 (Figure 2D). Moreover, stark differences were found in ASPR and ASDR with their corresponding EAPCs (Figure 3). For example, while South Asia and Southeast Asia are geographically close to East Asia, their ASPR in 2021 was lower than that of East Asia (Figure 3A). Notably, the ASPR of East Asia exhibited a declining trend according to the corresponding EAPC. With regard to DALYs, neither ASDR nor corresponding EAPC showed no significant differences across these three regions. The ASDR and the corresponding EAPC among these three regions had no large differences (Figure 3B). The EAPCs of ASPR and ASDR showed weak negative correlations with the SDI in 2021 (Figure 3). Notably, a significantly negative correlation was observed between the SDI levels and ASDR in 2021. A similar relationship between the SDI and the prevalence metrics was also found in 1990 (Figure S2).

**Figure 4:**
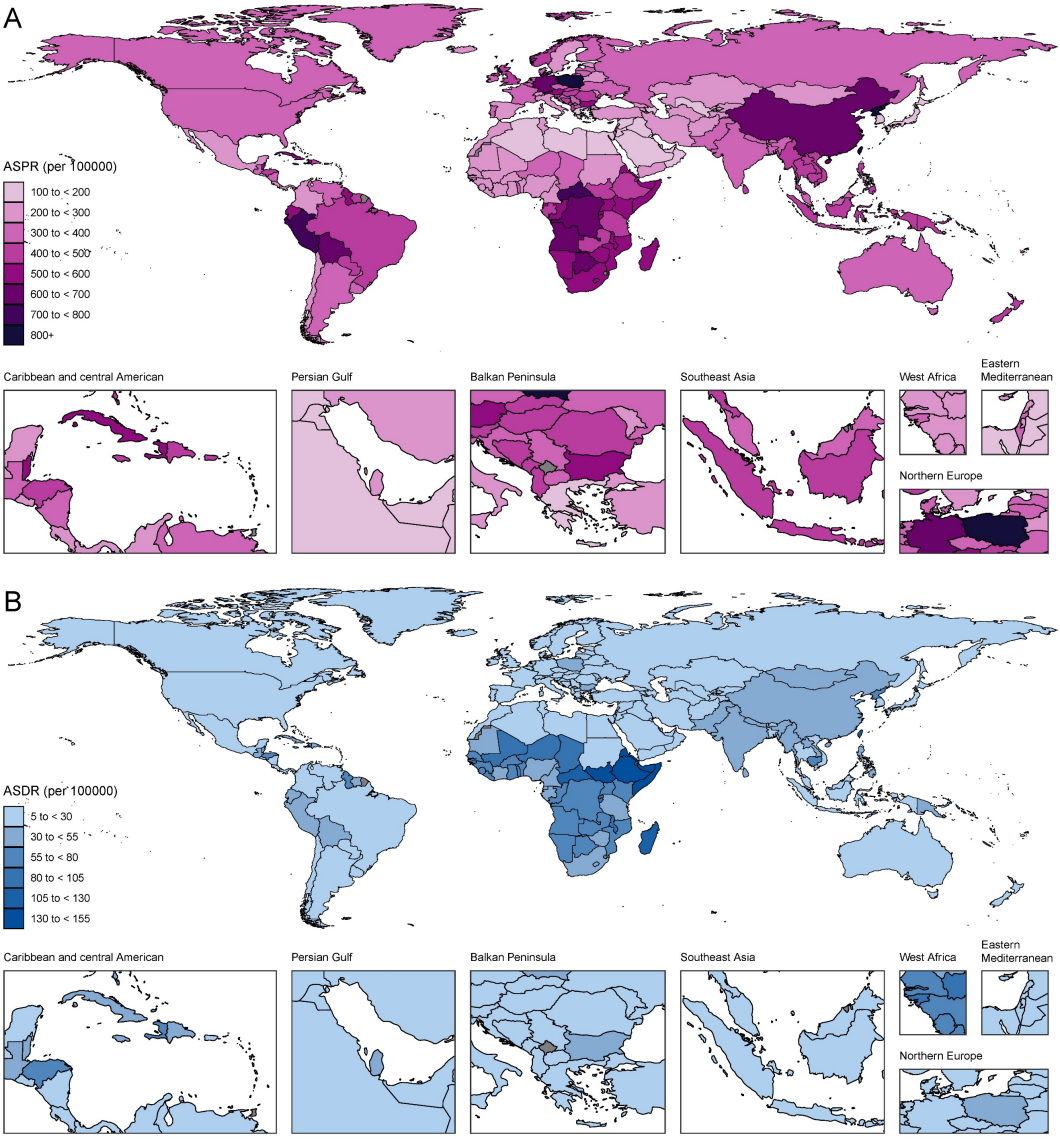
The ASPR and ASDR due to gastritis and duodenitis by country or region for male and female combined and all ages in 2021.

At the national and territorial levels, the highest ASPR of gastritis and duodenitis in 2021 was observed in certain countries in East Asia (e.g., China especially China's Taiwan, North Korea), Andean Latin America (e.g., Peru and Bolivia), Southern Sub-Saharan Africa (e.g., Central African Republic, Democratic Republic of the Congo, Botswana, Angola), Central Europe (e.g., Poland), Caribbean (e.g., Trinidad and Tobago, Saint Vincent and the Grenadines), which was consistent with the observations at the regional level (Figure 4, Table S1). Among them, China's Taiwan had the highest ASPR [2779.71 (95% UI: 2288.29-3331.72) per 100,000], followed by Republic of Korea (1114.91 [95% UI: 909.13-1376.32] per 100,000). In 2021, the ASPR of China's Taiwan was 22.3 times as high as the lowest ASPR observed in Japan (124.66 [95% UI: 101.47-152.71] per 100,000). Similar patterns were observed in ASIR with some minor change in rank order (Figure S3A). Although East Asia (China, China's Taiwan, Democratic People's Republic of Korea) is geographically close to High-income Asia Pacific (Japan, Republic of Korea), stark differences were found in ASPR and ASIR. These disparities might be associated with the SDI level and dietary habits. Because of higher ASPR and ASIR, countries and territories in those regions had higher ASDR and ASMR (Figure 4B, Figure S3B). The highest ASDR in 2021 was observed in Sub-Saharan Africa especially Eastern sub-Saharan Africa, including Ethiopia (147.63 [95% UI: 73.14-249.57] per 100,000), Somalia (143.12 [95% UI: 72.28-342.55] per 100,000), South Sudan (135.55 [95% UI: 68.32-252.47] per 100,000), which suggest low quality of life and poor survival for patients with gastritis and duodenitis.

### Global trends and their projections by age and sex

**Figure 5:**
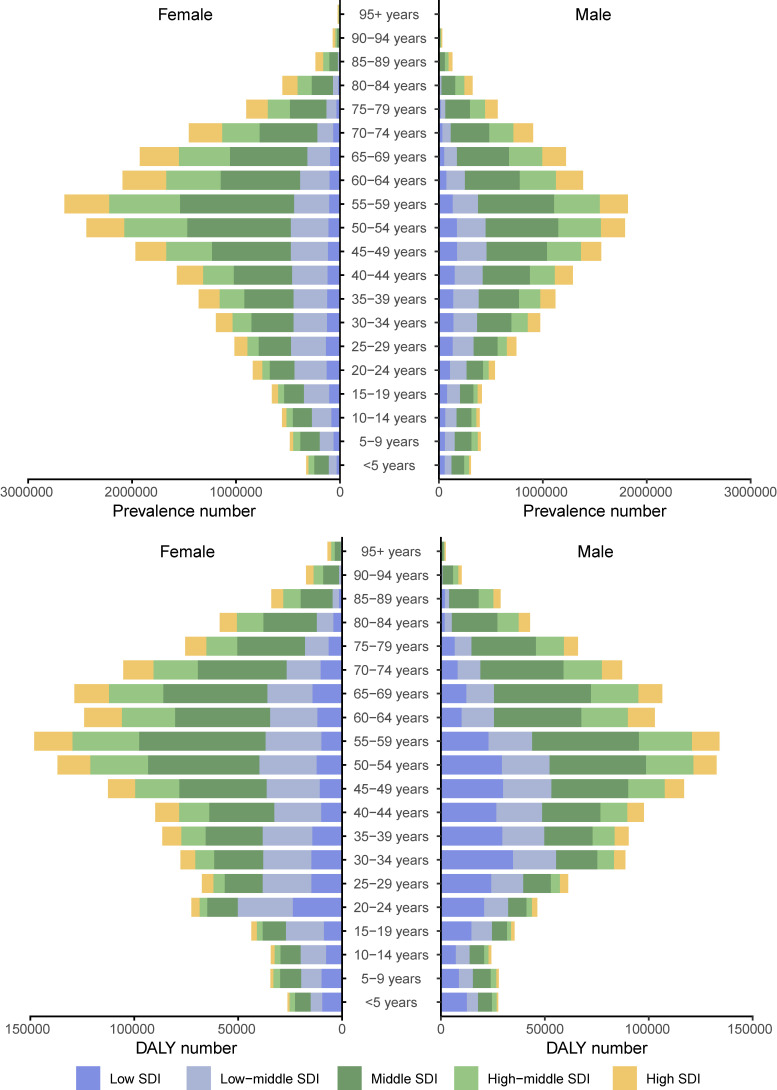
Distribution of prevalent cases due to gastritis and duodenitis by sex, age group, and SDI in 2021.

We further analyzed the global trends by age subgroup, sex, and SDI level. In 2021, the number of prevalent cases due to gastritis and duodenitis gradually increased with age, peaking at 55-59 years (Figure 5). Although the number of prevalent cases due to gastritis and duodenitis was greatest in the fifth decade of life, there were SDI level-specific variations in terms of which age subgroups carried the greatest disease burden. In all age subgroups, the number of prevalent cases in females surpassed that of males. Similarly, the number of DALYs showed a progressive increase with the age, peaking at 55-59 years (Figure 5, Table S2). In contrast to the distribution of prevalent cases, the number of DALYs between females and males were similar. At 55-59 years, the number of global DALYs in females slightly exceeded that of males, while this trend reversed in low SDI regions where male DALYs burden was comparatively higher.

**Figure 6:**
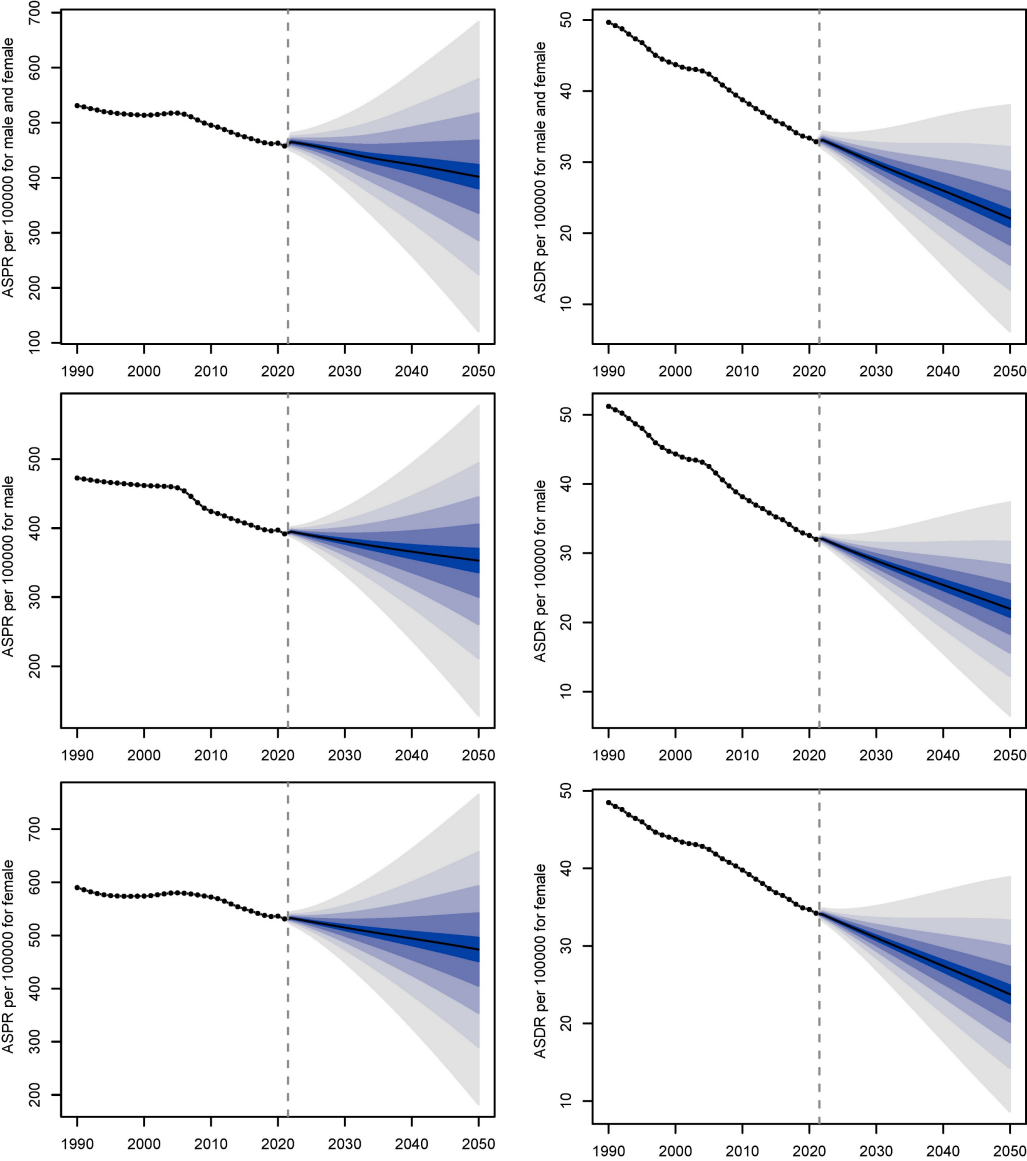
The projected change in ASPR (left) and ASDR (right) of gastritis and duodenitis from 2021 to 2050 by sex.

Next, a decomposition analysis by age subgroup and age projected to global trends from 2021 to 2050. In 2050, there will be approximately 51,244,723 individuals worldwide with gastritis and duodenitis (Figure 6). Between 2021 to 2050, the global ASPR of gastritis and duodenitis is projected to demonstrate a slight decline for both males and females, decreasing by 11.48% from about 454.31 per 100,000 in 2021 to about 402.14 per 100,000 in 2050. The global ASDR of gastritis and duodenitis is projected to decrease by 34.24% between 2021 and 2050, declining from about 33.59 per 100,000 in 2021 to 22.09 per 100,000 in 2050, indicating an underlying improvement in prevention and management of gastritis and duodenitis.

## Discussion

This report presents the most up-to-date and comprehensive assessment of gastritis and duodenitis in terms of ASIR, ASPR, ASMR, and ASDR at the global, regional, and national levels. Globally, the ASPR, ASIR, ASMR, and ASDR of gastritis and duodenitis decreased from 1990 to 2021. This trend aligns with existing epidemiological studies 10, 20, which attribute the reduction primarily to advancements in socioeconomic development and environmental conditions, such as SDI. While there was a global decrease in ASPR of gastritis and duodenitis between 1990 and 2021, regional disparities based on the SDI were observed. From 1990 to 2021, the ASPR and ASIR increased in low and low-middle SDI regions, while they decreased in middle, high-middle, and high SDI regions. Notably, the highest ASPR and ASIR were observed in middle SDI regions, whereas the lowest ASPR and ASIR were found in low SDI regions. This difference likely results from the epidemiological transition characteristic of middle SDI regions, where populations actively seek health check-ups but face an underdeveloped healthcare system. In 2021, the burden of gastritis and duodenitis was higher in females than in males across all age subgroups. Higher ASPR and prevalence number of gastritis and duodenitis were seen in the middle-aged individuals, which might be attributed to *H. pylori* infection and colonization, as well as poor dietary habits. Our forecasts suggest that the number of prevalent cases of gastritis and duodenitis is projected to increase by 33.82%, from 38.29 million in 1990 to 51.24 million in 2050. This growth is primarily attributed to population growth and aging.

At the regional level, the burden of gastritis and duodenitis was estimated from 1990 to 2021. Variations among different regions can be partially attributed to genetic effects and socioeconomic levels. For example, the highest ASPR was observed in East Asia, Andean Latin America, and Southern Sub-Saharan Africa, while the High-income Asia Pacific, Central Asia, North Africa and Middle East had the lowest ASPR in 2021. Geographically, East Asia, with the highest ASPR, is close to High-income Asia Pacific with the lowest ASPR, which might be attributed to socioeconomic developments and public health policies. In 2021, although the SDI in East Asia is close to that of North Africa and Middle East, there was a significant difference in ASPR between these two regions. This difference might be due to genetic variations and dietary habits. Gastritis and duodenitis are considered as precancerous lesions of gastric cancer 21, such as chronic atrophic gastritis 8 and intestinal metaplasia 9. Previous studies have suggested the existence of a distinct subclass of gastric cancer with clear alcohol-associated mutation signature and strong Asian specificity, almost all of which were attributable to alcohol intake behavior, smoking habit, and Asian-specific defective ALDH2 allele 22. Moreover, the SDI level is a crucial factor for evaluating the disease burden. Therefore, we analyzed the correlations between the SDI level with ASPR, ASIR, ASMR, and ASDR across 21 GBD regions in 1990 and 2021. We found a significantly negative correlation between SDI level and ASDR, but not between SDI level and ASPR, suggesting that the SDI level could be a more reliable index to measure and forecast ASDR. However, several studies employed a regression model to forecast prevalence with the SDI as a predictor because it is deemed to be closely tied to health outcomes 15, 23-25. Our analysis in disease burden of gastritis and duodenitis argued that a regression analysis of published data is needed before forecasting prevalence with the SDI as a predictor. Therefore, the GBD is capable of collecting more indices to adjust SDI for the modelling process.

There are several exposures to risk factors responsible for increasing ASPR and ASDR of gastritis and duodenitis, such as *H. pylori* infection 26, smoking 27, excessive alcohol consumption 27, poor dietary habits (e.g., sweet, spicy, cold, and fried foods) 28, low vegetables and fruits consumption 29, 30. East Asia and Sub-Saharan Africa regions exhibited the highest ASPR due to *H. pylori* infection, where a higher prevalence of *H. pylori* infection was reported 31. Furthermore, we observed a decline in the prevalence of gastritis and duodenitis from 1990 to 2021, along with a decline in the prevalence of *H. pylori* infection 32, 33. In low SDI regions, the *H. pylori* infection is associated with various factors 10, including limited awareness of gastritis and duodenitis 34, drinking water 35, low vegetables and fruits consumption 36. Additionally, the use of specific pharmacologic agents, particularly nonsteroidal anti-inflammatory drugs 37, represents a significant etiological factor in drug-induced gastrointestinal mucosal injury. Accordingly, evidence-based dietary interventions, such as cranberry juice, honey, and ginger, have demonstrated mucosal protective effects 38-40. A detailed investigation is necessary to determine the casual factors (e.g., population genetic differences and dietary habits) influencing the prevalence in certain regions, particularly East Asia, Sub-Saharan Africa, and Andean Latin America. While the SDI is an important index for describing the ASPR and ASDR, our findings suggest that the SDI is more applicable for describing and predicting ASDR.

Our forecast model shows that the estimated prevalence number of gastritis and duodenitis could reach up to 51.24 million by 2050. Because of weak correlations between SDI and ASPR, caution is necessary when considering the ASPR projection using SDI as a predictor. Currently, there is no feasible model to adjust the impact of COVID-19 on forecast estimates for gastritis and duodenitis. On the one hand, COVID-19 may cause the digestive symptoms 41-43, potentially contributing to the rising ASPR observed in certain regions. On the other hand, the preventive and management strategies during the COVID-19 pandemic leads to statistical bias in epidemiological surveys of gastritis and duodenitis. From 2020 to 2021, the ASPR and ASIR in the high SDI regions increased, while the low and low-middle SDI regions exhibited an unusual decline (Figure 1). This unexpected change might be explained by the pandemic-induced shifts in public health policies, further leading to regional disparities in gastritis and duodenitis. Furthermore, this forecast is based on age-period-cohort (APC) model rather than various risk factors in that the forecast model cannot be yet based on forecasted rates of risk factors. Countries and territories with higher prevalence urgently need to establish programs for early detection and treatment of gastritis and duodenitis to alleviate the burden.

Although we present, to our knowledge, the most up-to-date and comprehensive assessment of the global temporal trends of gastritis and duodenitis across 204 countries and territories over three decades, there are some considerable limitations to interpret these data and findings. Gastritis and duodenitis are highly prevalent inflammatory conditions. Chronic gastritis (particularly subtypes such as chronic atrophic gastritis and intestinal metaplasia) is associated with an increased risk of gastric cancer 44. Our analysis inherits the GBD 2021's conceptual limitation of conflating etiologically distinct subtypes under ICD-10 K29, which precludes differentiation between gastritis and duodenitis subtypes, such as *H. pylori* infection 45-47. Future iterations of the GBD should prioritize coding reforms to disentangle these entities, as their merged analysis may attenuate causal inference for malignancy risk. The estimated prevalence has wide uncertainty intervals in almost all regions in GBD 2021, indicating higher uncertainty in the estimates 24. Another limitation is the lack of risk factors for gastritis and duodenitis in GBD 2021, such as dietary habits (e.g., vegetables and fruits, vitamin A, and salt consumption), smoking, alcohol consumption, and nonsteroidal anti-inflammatory drug abuse. However, several known causal risk factors for gastritis and duodenitis have not been evaluated in GBD 2021. Besides, there is a lack of finer variations within countries, especially in vast countries including China and Russia. Therefore, more detailed geographical data and risk factors further need to be collected.

## Conclusion

In conclusion, despite a global decrease in age-standardized rates of gastritis and duodenitis over the past three decades and an expected decline in the next three decades, the absolute number of new cases is rising due to population growth and aging from 1990 to 2021. Our analysis reveals significant variations in the burden of gastritis and duodenitis across regions, countries and territories. Our findings underscore the importance of active surveillance and prevention of gastritis and duodenitis as the population ages, particularly in the elderly and among females. Future research should focus on understanding the complex interplay of genetic, environmental, and lifestyle factors contributing to gastritis and duodenitis development. Disease prevention requires established public policy interventions to control risk factors and promote scientific awareness.

## Supplementary Material

Supplementary figures and tables.
