# Supplementary material for: Global, regional, and national burden of gastritis and duodenitis from 1990 to 2021 with projections to 2050: a systematic analysis of the Global Burden of Disease Study 2021
Source: Int J Med Sci. 2025 May 10;22(11):2570–82. doi: 10.7150/ijms.109762 (PMC12163426; doi:10.7150/ijms.109762)
Supplement: Supplementary file 1 — Supplementary figures and tables. [file ijmsv22p2570s1.pdf]

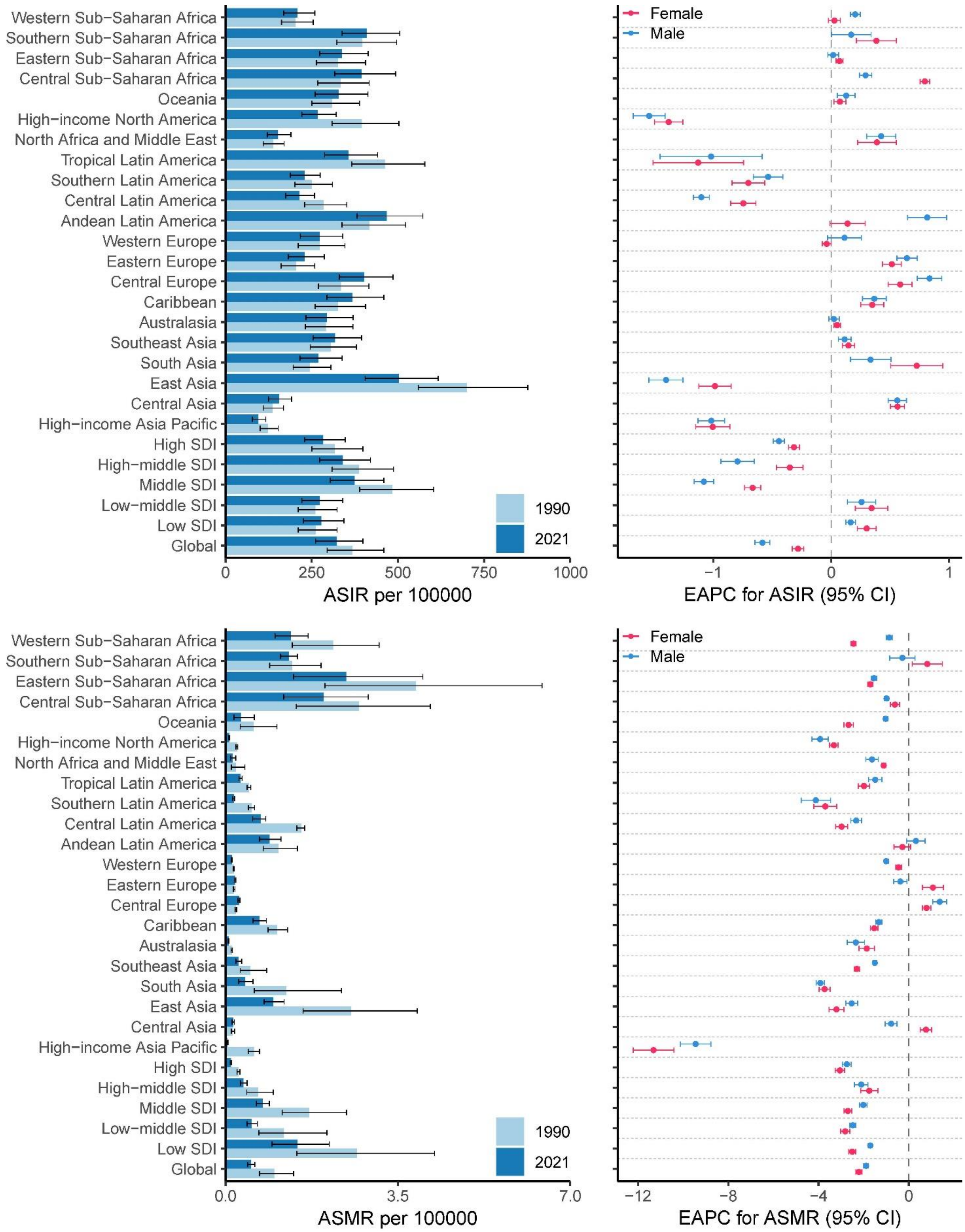

Figure S1 The ASIR and ASMR due to gastritis and duodenitis between 1990 and 2021, and the corresponding EAPC by sex from 1990 to 2021.

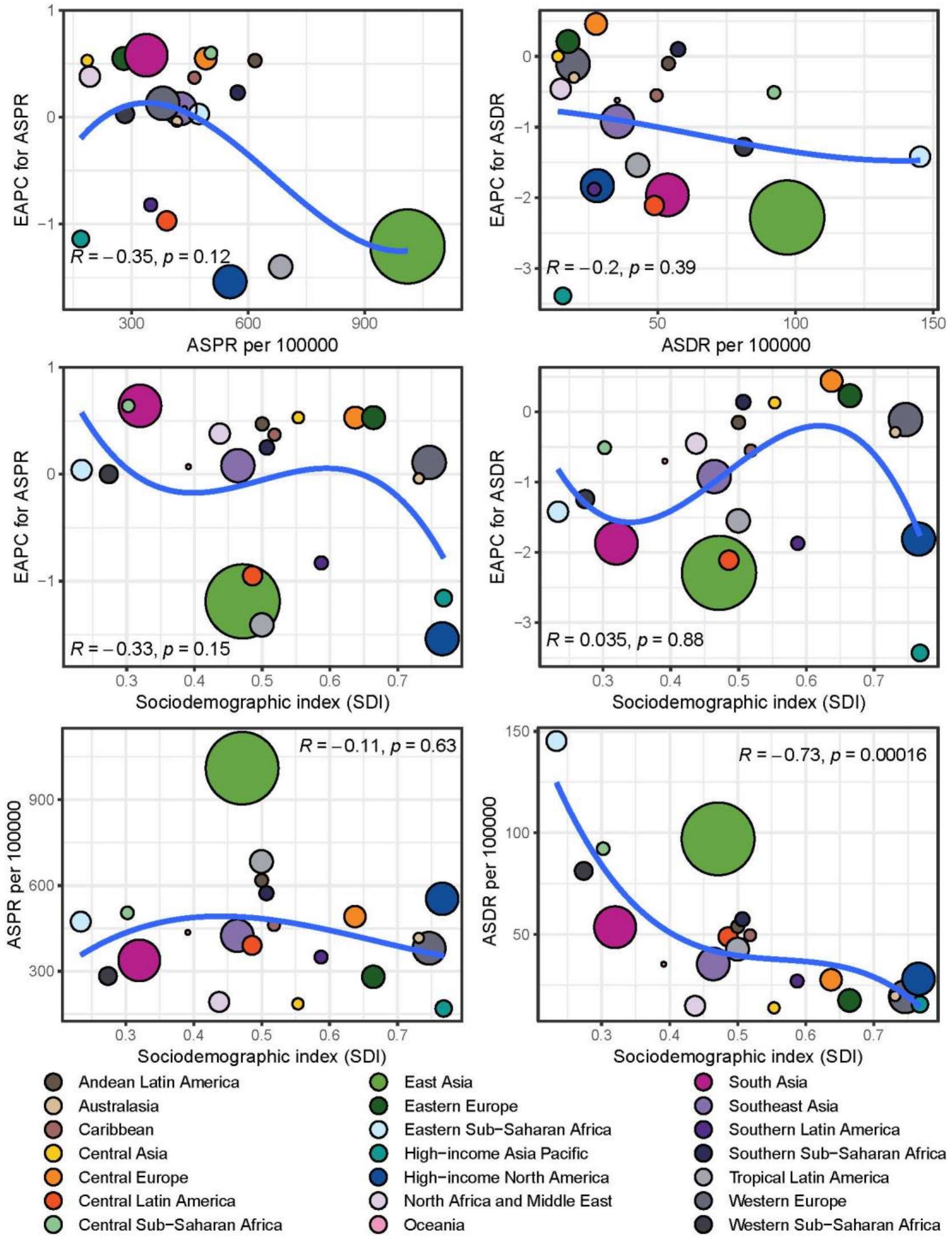

Figure S2 The relationships of EAPC and SDI with ASPR and ASDR among different regions in 1990. The size of points represents the corresponding number of prevalent cases and DALYs.

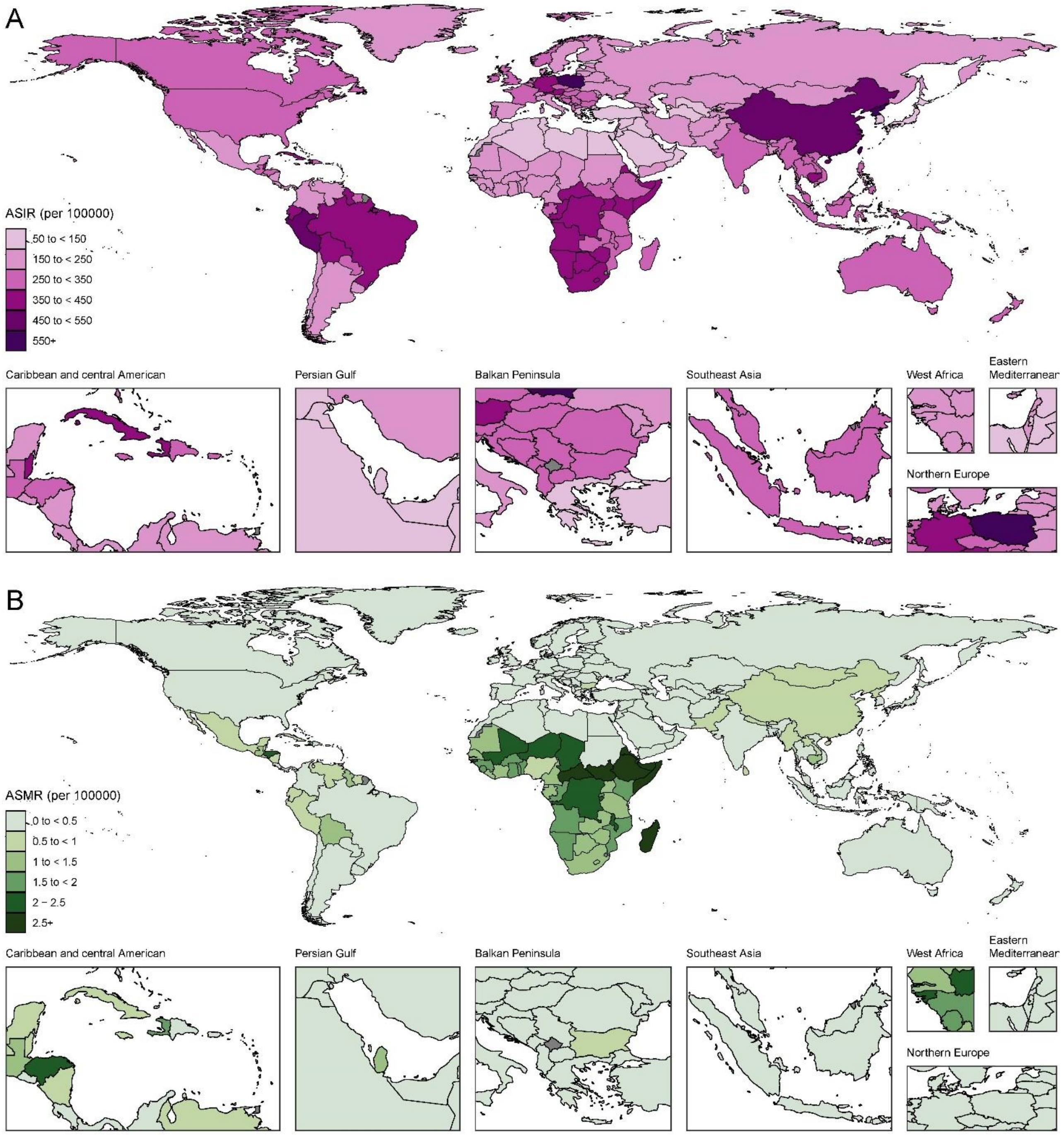

Figure S3 The ASIR and ASMR due to gastritis and duodenitis by country or region for male and female combined and all ages in 2021.

Table S1 The global and regional incidence, prevalence, mortality, and DALYs due to gastritis and duodenitis between 1990 and 2021, as well as the EAPC from 1990 to 2021.

	1990		2021		1990-2021
	Cases number *10^5 (95% UI)	ASR per 100,000 (95% UI)	Cases number *10^5 (95% UI)	ASR per 100,000 (95% UI)	EAPC (95% CI)
**Incidence**					
Socio-demographic index					
High SDI	31.30[24.68-39.21]	316.97[250.23-398.42]	41.00[32.69-49.83]	283.45[229.9-347.83]	-0.39[-0.43 to -0.35]
High-middle SDI	40.86[32.59-51.27]	387.34[308.64-486.89]	57.56[45.5-71.88]	340.00[273-420.49]	-0.55[-0.67 to -0.42]
Middle SDI	67.50[55.1-83.63]	483.71[388.92-604.26]	101.28[81.37-125.76]	374.18[304.32-459.58]	-0.85[-0.92 to -0.78]
Low-middle SDI	24.06[19.55-29.96]	260.44[211.07-323.45]	49.24[39.55-62.11]	273.54[221.41-340.43]	0.32[0.19 to 0.45]
Low SDI	9.26[7.54-11.48]	261.59[211.35-323.69]	22.70[18.41-28.17]	277.82[226.19-342.9]	0.24[0.18 to 0.3]
Region					
Andean Latin America	1.23[0.98-1.52]	417.27[338.02-522.81]	3.02[2.47-3.72]	467.34[381.61-572.04]	0.40[0.25 to 0.54]
Australasia	0.64[0.51-0.81]	291[232.09-369.44]	1.09[0.87-1.36]	293.73[233.4-370.02]	0.03[0 to 0.06]
Caribbean	0.98[0.8-1.23]	325.63[259.76-406.31]	1.90[1.51-2.39]	367.54[293.9-459.49]	0.36[0.26 to 0.46]
Central Asia	0.82[0.67-1.02]	136.53[109.13-168.18]	1.49[1.19-1.86]	155.12[124.6-191.52]	0.53[0.47 to 0.6]
Central Europe	4.58[3.64-5.68]	334.80[269.12-416.16]	6.35[5.12-7.68]	402.77[330.65-485.85]	0.67[0.57 to 0.76]
Central Latin America	3.38[2.76-4.17]	284.04[229.27-351.71]	5.53[4.48-6.73]	214.68[174.9-257.9]	-0.90[-0.99 to -0.82]
Central Sub-Saharan Africa	1.12[0.92-1.4]	333.48[267.22-416.79]	3.39[2.75-4.22]	394.63[317.06-493.73]	0.59[0.56 to 0.62]
East Asia	74.28[60-93.41]	699.77[559.15-876.88]	100.17[79.36-124.79]	502.37[404.99-617]	-1.18[-1.32 to -1.04]
Eastern Europe	5.18[4.09-6.55]	204.38[161.49-258.75]	5.97[4.68-7.58]	228.76[182.37-286.63]	0.56[0.48 to 0.64]
Eastern Sub-Saharan Africa	3.81[3.12-4.68]	326.53[263.56-406]	9.20[7.57-11.32]	337.68[273.5-413.6]	0.05[0.01 to 0.09]
High-income Asia Pacific	2.29[1.85-2.86]	123.74[100.81-152.67]	2.00[1.62-2.45]	95.32[78.38-116.62]	-1.04[-1.17 to -0.91]

High-income North America	12.42[9.72-15.64]	395.31[309.18-502.92]	12.77[10.42-15.26]	266.80[221.48-321.45]	-1.47[-1.59 to -1.34]
North Africa and Middle East	3.84[3.1-4.82]	137.34[109.55-169.76]	9.16[7.27-11.49]	152.40[121.56-189.44]	0.40[0.25 to 0.54]
Oceania	0.17[0.14-0.21]	309.67[250.26-388.33]	0.41[0.33-0.51]	327.78[260.26-413.2]	0.09[0.04 to 0.15]
South Asia	22.07[17.72-27.53]	243.83[196.13-306.18]	49.07[39.41-62.55]	269.29[215.65-338.44]	0.60[0.4 to 0.8]
Southeast Asia	12.56[10.21-15.53]	305.66[245.97-379.73]	23.12[18.28-29.07]	317.23[253.98-394.4]	0.13[0.08 to 0.18]
Southern Latin America	1.20[0.97-1.49]	249.87[201.27-310.38]	1.70[1.39-2.05]	228.66[188.27-274.39]	-0.64[-0.77 to -0.5]
Southern Sub-Saharan Africa	1.56[1.25-1.96]	397.28[322.76-496.17]	3.01[2.44-3.73]	410.06[338.31-505.6]	0.30[0.14 to 0.47]
Tropical Latin America	5.44[4.35-6.87]	461.86[365.84-578.04]	8.99[7.2-11.18]	356.95[287.79-441.41]	-1.09[-1.5 to -0.69]
Western Europe	12.72[9.86-16.05]	273.09[210.95-346.12]	16.35[12.81-20.38]	272.75[216.99-339.95]	0.01[-0.07 to 0.08]
Western Sub-Saharan Africa	2.84[2.32-3.52]	202.55[162.3-254.18]	7.34[5.99-9.14]	208.68[168.89-259.1]	0.09[0.05 to 0.12]
Global	173.14[139.59-215.73]	367.51[295.09-459.29]	272.03[218.51-336.48]	323.24[261.35-398.64]	-0.42[-0.47 to -0.37]
**Prevalence**					
Socio-demographic index					
High SDI	44.71[35.23-55.51]	451.17[356.99-558.5]	60.36[50.03-73.81]	413.52[342.32-492.22]	-0.23[-0.27 to -0.19]
High-middle SDI	56.88[45.7-71.18]	539.47[432.24-672.09]	79.72[63.91-98.44]	467.48[380.05-572.29]	-0.60[-0.73 to -0.46]
Middle SDI	96.02[78.38-117.78]	696.36[561.66-864.56]	141.74[114.63-175.93]	523.25[428.09-643.39]	-0.95[-1.03 to -0.86]
Low-middle SDI	33.54[26.86-41.66]	367.50[296.99-452.17]	69.00[55.4-85.35]	384.61[309.45-469.52]	0.33[0.18 to 0.48]
Low SDI	13.01[10.61-15.97]	374.17[304.1-461.93]	31.79[25.71-38.88]	395.12[319.81-486.35]	0.24[0.17 to 0.3]
Region					
Andean Latin America	1.80[1.45-2.23]	617.80[497.85-753.24]	4.54[3.73-5.48]	705.21[582.53-847.45]	0.47[0.28 to 0.66]
Australasia	0.91[0.70-1.15]	416.54[321.16-524.06]	1.52[1.22-1.89]	408.74[324.13-513.08]	-0.04[-0.06 to -0.02]
Caribbean	1.38[1.12-1.67]	461.63[375.7-567.73]	2.71[2.19-3.40]	523.76[423.94-650.40]	0.37[0.25 to 0.49]
Central Asia	1.12[0.90-1.37]	185.92[148.57-226.67]	2.02[1.61-2.51]	210.60[168.14-259.02]	0.53[0.47 to 0.59]
Central Europe	6.75[5.37-8.34]	491.17[391.21-594.59]	9.01[7.56-10.85]	561.67[472.68-669.36]	0.53[0.43 to 0.63]

Central Latin America	4.59[3.72-5.66]	390.55[316.22-486.73]	7.44[6.14-9.22]	288.82[238.00-353.72]	-0.95[-1.04 to -0.87]
Central Sub-Saharan Africa	1.65[1.36-2.02]	504.23[409.63-623.32]	5.00[4.11-6.14]	603.80[493.07-749.34]	0.64[0.60 to 0.67]
East Asia	106.12[84.89-131.72]	1010.31[807.96-1266.14]	144.85[116.60-178.64]	721.66[594.36-875.06]	-1.19[-1.34 to -1.05]
Eastern Europe	7.16[5.69-8.96]	280.34[222.85-350.90]	8.22[6.52-10.41]	310.69[247.26-390.86]	0.53[0.45 to 0.62]
Eastern Sub-Saharan Africa	5.37[4.40-6.52]	473.37[383.20-591.00]	12.84[10.49-15.69]	484.05[394.98-597.45]	0.04[0.00 to 0.08]
High-income Asia Pacific	3.16[2.49-3.87]	169.43[134.50-208.33]	2.68[2.17-3.25]	127.33[102.67-156.54]	-1.16[-1.30 to -1.01]
High-income North America	17.41[13.57-21.94]	553.38[426.43-703.83]	17.28[14.43-20.77]	359.62[300.59-428.00]	-1.54[-1.67 to -1.40]
North Africa and Middle East	5.34[4.27-6.62]	192.59[153.33-236.09]	12.69[10.03-15.67]	211.75[169.7-257.89]	0.38[0.20 to 0.56]
Oceania	0.23[0.19-0.29]	436.03[348.08-540.52]	0.57[0.45-0.70]	458.80[366.63-572.32]	0.07[-0.01 to 0.15]
South Asia	30.33[24.07-38.2]	337.84[272.24-418.86]	68.21[54.26-86.33]	373.67[298.46-460.68]	0.64[0.41 to 0.87]
Southeast Asia	17.39[13.99-21.47]	424.56[342.64-523.00]	31.67[25.2-39.74]	434.57[349.06-539.02]	0.08[0.02 to 0.15]
Southern Latin America	1.68[1.35-2.07]	349.31[279.49-428.00]	2.28[1.89-2.75]	306.86[253.58-365.95]	-0.83[-0.97 to -0.68]
Southern Sub-Saharan Africa	2.23[1.81-2.75]	573.03[465.86-703.53]	4.22[3.42-5.22]	576.02[468.26-711.16]	0.25[0.04 to 0.45]
Tropical Latin America	7.96[6.44-9.74]	683.81[557.35-835.42]	12.33[10.08-15.21]	488.62[399.91-597.39]	-1.41[-1.78 to -1.03]
Western Europe	17.85[13.93-22.51]	380.32[299.19-479.10]	22.95[18.41-28.62]	376.78[303.96-461.43]	0.11[-0.05 to 0.27]
Western Sub-Saharan Africa	3.91[3.16-4.79]	283.17[228.86-348.59]	9.91[7.99-12.11]	284.83[232.85-349.79]	0.00[-0.03 to 0.04]
Global	244.37[197.32-300.46]	521.67[419.28-647.52]	382.93[311.67-472.26]	454.31[372.66-558.27]	-0.43[-0.49 to -0.37]
**Deaths**					
Socio-demographic index					
High SDI	0.03[0.03-0.03]	0.26[0.23-0.29]	0.03[0.02-0.03]	0.11[0.10-0.12]	-2.86[-3.05 to -2.67]
High-middle SDI	0.06[0.04-0.09]	0.66[0.43-0.97]	0.07[0.06-0.08]	0.36[0.30-0.44]	-1.93[-2.25 to -1.60]

Middle SDI	0.15[0.10-0.22]	1.69[1.15-2.46]	0.18[0.15-0.21]	0.75[0.63-0.89]	-2.37[-2.53 to -2.21]
Low-middle SDI	0.08[0.04-0.13]	1.19[0.68-2.06]	0.07[0.06-0.09]	0.52[0.44-0.65]	-2.65[-2.81 to -2.48]
Low SDI	0.07[0.04-0.11]	2.66[1.45-4.24]	0.08[0.05-0.12]	1.45[0.94-2.11]	-2.05[-2.17 to -1.93]
Region					
Andean Latin America	0.00[0.00-0.00]	1.08[0.77-1.46]	0.01[0.00-0.01]	0.89[0.69-1.12]	-0.02[-0.40 to 0.37]
Australasia	0.00[0.00-0.00]	0.13[0.11-0.14]	0.00[0.00-0.00]	0.07[0.06-0.07]	-2.07[-2.33 to -1.81]
Caribbean	0.00[0.00-0.00]	1.05[0.86-1.26]	0.00[0.00-0.00]	0.69[0.56-0.83]	-1.42[-1.56 to -1.28]
Central Asia	0.00[0.00-0.00]	0.14[0.12-0.18]	0.00[0.00-0.00]	0.15[0.13-0.18]	0.08[-0.12 to 0.28]
Central Europe	0.00[0.00-0.00]	0.21[0.19-0.23]	0.01[0.01-0.01]	0.27[0.25-0.30]	1.13[0.89 to 1.37]
Central Latin America	0.01[0.01-0.01]	1.54[1.45-1.61]	0.02[0.01-0.02]	0.71[0.55-0.82]	-2.66[-2.91 to -2.41]
Central Sub-Saharan Africa	0.01[0.00-0.01]	2.71[1.44-4.16]	0.01[0.01-0.01]	1.99[1.18-2.9]	-0.87[-1.00 to -0.74]
East Asia	0.17[0.10-0.26]	2.55[1.57-3.90]	0.18[0.15-0.23]	0.97[0.78-1.19]	-2.86[-3.14 to -2.58]
Eastern Europe	0.00[0.00-0.01]	0.17[0.16-0.19]	0.01[0.01-0.01]	0.19[0.18-0.21]	0.21[-0.03 to 0.46]
Eastern Sub-Saharan Africa	0.04[0.02-0.06]	3.87[2.02-6.43]	0.05[0.03-0.08]	2.45[1.38-4.01]	-1.62[-1.74 to -1.50]
High-income Asia Pacific	0.01[0.01-0.01]	0.58[0.46-0.69]	0.00[0.00-0.00]	0.03[0.02-0.05]	-10.56[-11.39 to -9.73]
High-income North America	0.01[0.01-0.01]	0.23[0.21-0.25]	0.01[0.00-0.01]	0.08[0.07-0.08]	-3.59[-3.85 to -3.32]
North Africa and Middle East	0.00[0.00-0.01]	0.21[0.12-0.39]	0.01[0.00-0.01]	0.14[0.10-0.21]	-1.37[-1.51 to -1.23]
Oceania	0.00[0.00-0.00]	0.57[0.30-1.05]	0.00[0.00-0.00]	0.31[0.17-0.59]	-2.27[-2.42 to -2.12]
South Asia	0.07[0.04-0.13]	1.23[0.59-2.36]	0.05[0.04-0.08]	0.40[0.27-0.56]	-3.74[-3.95 to -3.52]
Southeast Asia	0.01[0.01-0.02]	0.50[0.3-0.83]	0.02[0.01-0.02]	0.26[0.21-0.33]	-1.99[-2.06 to -1.92]
Southern Latin America	0.00[0.00-0.00]	0.53[0.46-0.59]	0.00[0.00-0.00]	0.17[0.15-0.18]	-3.94[-4.52 to -3.35]
Southern Sub-Saharan Africa	0.00[0.00-0.00]	1.35[0.9-1.94]	0.01[0.01-0.01]	1.29[1.12-1.47]	0.37[-0.25 to 0.98]

Tropical Latin America	0.00[0.00-0.00]	0.47[0.44-0.51]	0.01[0.01-0.01]	0.31[0.27-0.34]	-1.73[-2.00 to -1.46]
Western Europe	0.01[0.01-0.01]	0.17[0.16-0.18]	0.01[0.01-0.01]	0.13[0.11-0.14]	-0.66[-0.77 to -0.56]
Western Sub-Saharan Africa	0.02[0.01-0.03]	2.19[1.36-3.12]	0.03[0.02-0.04]	1.32[1.01-1.68]	-1.50[-1.60 to -1.39]
Global	0.38[0.26-0.53]	0.99[0.69-1.38]	0.43[0.37-0.49]	0.52[0.45-0.59]	-2.03[-2.15 to -1.92]
**DALYs**					
Socio-demographic index					
High SDI	2.42[1.75-3.26]	24.16[17.31-32.76]	2.89[2.04-3.95]	19.24[13.15-26.71]	-0.66[-0.73 to -0.60]
High-middle SDI	3.99[2.84-5.41]	38.83[27.67-52.41]	4.64[3.44-6.19]	26.6[19.51-35.70]	-1.33[-1.48 to -1.17]
Middle SDI	8.72[6.44-11.54]	69.74[51.20-92.83]	9.89[7.71-12.91]	37.44[29.35-48.56]	-1.92[-2.00 to -1.83]
Low-middle SDI	4.50[3.06-6.34]	52.92[35.06-77.79]	5.73[4.46-7.35]	33.18[25.96-42.07]	-1.49[-1.60 to -1.38]
Low SDI	3.38[2.17-4.92]	100.61[61.62-148.55]	4.95[3.39-6.78]	63.44[44.08-87.16]	-1.59[-1.68 to -1.51]
Region					
Andean Latin America	0.15[0.11-0.19]	53.87[41.54-68.58]	0.30[0.23-0.39]	47.79[37.19-61.58]	-0.15[-0.35 to 0.05]
Australasia	0.04[0.03-0.06]	19.6[13.49-27.42]	0.07[0.05-0.09]	17.73[11.55-25.22]	-0.29[-0.32 to -0.26]
Caribbean	0.15[0.12-0.18]	49.56[39.54-60.68]	0.22[0.17-0.28]	41.87[32.42-53.34]	-0.55[-0.61 to -0.49]
Central Asia	0.08[0.06-0.11]	13.79[10.58-18.21]	0.14[0.10-0.18]	14.44[10.87-19.28]	0.13[0.05 to 0.21]
Central Europe	0.38[0.27-0.52]	27.61[19.79-37.45]	0.50[0.38-0.66]	30.47[22.26-40.74]	0.44[0.33 to 0.55]
Central Latin America	0.50[0.43-0.59]	48.77[42.81-56.37]	0.68[0.53-0.83]	26.66[20.93-32.61]	-2.11[-2.21 to -2.02]
Central Sub-Saharan Africa	0.29[0.19-0.41]	92.18[60.57-131.63]	0.58[0.40-0.79]	75.76[53.51-100.54]	-0.51[-0.60 to -0.43]
East Asia	9.17[6.4-12.55]	96.99[67.73-132.85]	9.45[7.29-12.49]	47.09[36.02-62.08]	-2.29[-2.45 to -2.14]
Eastern Europe	0.45[0.34-0.6]	17.45[13.20-23.23]	0.51[0.39-0.68]	18.71[13.93-24.81]	0.23[0.10 to 0.36]
Eastern Sub-Saharan Africa	1.70[1.05-2.71]	145.22[86.84-229.21]	2.67[1.64-4.02]	97.53[61.07-145.49]	-1.42[-1.52 to -1.32]
High-income Asia Pacific	0.28[0.22-0.34]	15.54[12.34-19.19]	0.15[0.10-0.2]	6.04[4.02-8.48]	-3.43[-3.85 to -3.00]

High-income North America	0.89[0.64-1.21]	28.05[19.92-38.05]	0.80[0.56-1.09]	16.44[11.31-22.58]	-1.81[-1.96 to -1.66]
North Africa and Middle East	0.39[0.27-0.54]	14.76[10.11-20.51]	0.73[0.52-0.99]	12.70[9.32-17.17]	-0.45[-0.57 to -0.33]
Oceania	0.02[0.01-0.03]	35.30[23.39-51.84]	0.03[0.02-0.05]	30.05[20.24-43.15]	-0.70[-0.76 to -0.64]
South Asia	4.29[2.70-6.38]	53.50[32.66-83.91]	5.26[3.87-6.98]	30.02[22.29-39.27]	-1.87[-2.00 to -1.74]
Southeast Asia	1.41[1.04-1.93]	35.39[25.77-48.11]	1.89[1.38-2.60]	26.68[19.77-36.37]	-0.92[-0.96 to -0.88]
Southern Latin America	0.13[0.10-0.16]	26.96[21.38-33.48]	0.12[0.09-0.17]	16.37[11.73-21.99]	-1.87[-2.07 to -1.68]
Southern Sub-Saharan Africa	0.21[0.16-0.27]	57.38[43.05-77.02]	0.35[0.28-0.44]	52.93[42.79-65.19]	0.14[-0.30 to 0.58]
Tropical Latin America	0.49[0.36-0.64]	42.68[31.92-56.2]	0.74[0.55-0.98]	29.35[21.6-38.71]	-1.55[-1.89 to -1.21]
Western Europe	0.92[0.65-1.26]	19.24[13.56-26.36]	1.13[0.80-1.54]	17.7[12.18-24.49]	-0.11[-0.25 to 0.03]
Western Sub-Saharan Africa	1.10[0.76-1.47]	81.24[54.79-111.77]	1.79[1.34-2.30]	54.05[41.4-68.99]	-1.24[-1.32 to -1.16]
Global	23.02[17.1-30.03]	50.42[37.54-65.63]	28.13[21.75-36.12]	33.59[25.96-43.11]	-1.30[-1.35 to -1.24]

Table S2 The global incidence, prevalence, mortality, and DALYs due to gastritis and duodenitis in 2021 by sex and age subgroup.

	Male		Female	
	Cases number (95% UI)	ASR per 100,000 (95% UI)	Cases number (95% UI)	ASR per 100,000 (95% UI)
**Incidence**				
<5 years	265036.32[373885.29-177851.27]	77.96[109.97-52.31]	284665.44[406506.05-186934.39]	89.46[127.75-58.75]
5-9 years	280686.17[435344.82-163294.96]	79.18[122.81-46.07]	345412.40[536914.65-197034.72]	103.86[161.44-59.24]
10-14 years	278443.72[421397.23-163883.03]	81.01[122.60-47.68]	393407.92[596493.10-228694.89]	121.83[184.72-70.82]
15-19 years	310710.85[470825.59-184749.22]	97.00[146.98-57.67]	467473.14[715928.38-279394.58]	153.95[235.77-92.01]
20-24 years	414700.86[616637.74-244999.50]	136.68[203.24-80.75]	594701.04[900338.33-346769.46]	202.45[306.5-118.05]
25-29 years	553036.99[882296.99-342196.2]	185.98[296.71-115.08]	710839.45[1139430.58-430549.31]	244.29[391.57-147.96]
30-34 years	708725.57[1163041.28-415897.97]	231.95[380.64-136.11]	850937.53[1405649.15-489255.90]	284.66[470.23-163.67]
35-39 years	823958.94[1402545.39-499892.35]	291.09[495.49-176.60]	984525.91[1718340.81-577470.38]	354.40[618.55-207.87]
40-44 years	963201.52[1533160.77-572940.54]	381.98[608.01-227.21]	1161786.59[1868937.26-674173.83]	468.29[753.33-271.75]
45-49 years	1143082.99[1829748.98-676431.09]	480.57[769.25-284.38]	1439797.37[2339934.65-827057.83]	611.01[993.00-350.98]
50-54 years	1296618.03[2096047.52-783629.55]	584.11[944.25-353.02]	1745812.83[2873539.41-1028107.14]	783.08[1288.91-461.15]
55-59 years	1279335.53[1926160.56-783283.88]	657.00[989.17-402.25]	1827165.40[2803164.58-1075673.82]	909.02[1394.58-535.15]
60-64 years	958576.2[1431296.37-610945.08]	616.30[920.23-392.80]	1420263.82[2145668.39-888905.88]	863.32[1304.27-540.33]
65-69 years	864391.44[1198783.13-552027.89]	655.67[909.32-418.73]	1327042.14[1862581.55-848151.92]	921.50[1293.38-588.96]
70-74 years	644716.45[908109.05-405143.53]	668.85[942.11-420.31]	990568.96[1406063.56-609322.93]	905.06[1284.69-556.72]
75-79 years	389869.24[585639.53-247818.16]	652.10[979.55-414.50]	612796.29[913486.79-389648.96]	849.95[1267.01-540.45]
80-84 years	206010.85[313393.4-136929.71]	562.07[855.05-373.60]	352758.15[530085.53-233544.43]	692.62[1040.79-458.55]
85-89 years	75639.09[103187.89-49443.78]	438.42[598.1-286.59]	145745.45[201978.62-93276.46]	511.94[709.47-327.64]
90-94 years	19638.73[27007.47-12605.37]	336.94[463.37-216.27]	50181.53[69056.48-32213.36]	416.07[572.57-267.09]
95+ years	4357.75[6719.57-2436.38]	288.20[444.4-161.13]	15905.21[24642.93-8948.64]	403.86[625.73-227.22]
**Prevalence**				
<5 years	308889.34[433384.77-214921.59]	90.86[127.47-63.22]	325058.90[462568.52-219455.97]	102.16[145.37-68.97]

5-9 years	403337.07[668040.37-240595.30]	113.78[188.46-67.87]	483535.68[801447.38-283792.65]	145.39[240.98-85.33]
10-14 years	393244.42[603424.74-220367.20]	114.41[175.56-64.11]	557655.11[855416.16-318639.31]	172.69[264.9-98.67]
15-19 years	413645.08[640796.44-252553.43]	129.13[200.04-78.84]	656534.94[1030644.05-393152.28]	216.21[339.42-129.48]
20-24 years	540018.92[854283.25-316321.07]	177.99[281.56-104.26]	840145.85[1379578.71-482286.04]	286.01[469.64-164.18]
25-29 years	745968.96[1198118.33-439853.74]	250.87[402.92-147.92]	1016181.99[1708123.1-576834.08]	349.22[587.01-198.23]
30-34 years	976516.86[1498182.4-585480.97]	319.59[490.32-191.62]	1194783.12[1871673.82-702589.54]	399.69[626.12-235.03]
35-39 years	1123204.56[1774481.57-670441.00]	396.80[626.88-236.85]	1360749.24[2156905.55-768604.33]	489.83[776.42-276.67]
40-44 years	1290450.23[2016487.78-782281.02]	511.76[799.68-310.23]	1571542.49[2484908.44-934388.22]	633.46[1001.62-376.63]
45-49 years	1563156.29[2484850.10-950432.29]	657.17[1044.66-399.57]	1969271.30[3140880.75-1140635.44]	835.70[1332.9-484.05]
50-54 years	1792830.57[3020528.00-1111878.99]	807.65[1360.72-500.89]	2442698.43[4071522.53-1459681.63]	1095.66[1826.27-654.73]
55-59 years	1821314.92[2727335.24-1127768.53]	935.33[1400.61-579.16]	2653933.88[4008137.61-1580178.53]	1320.34[1994.06-786.14]
60-64 years	1389133.51[2082106.99-896984.78]	893.12[1338.66-576.7]	2093935.94[3227517.72-1311012.65]	1272.82[1961.89-796.92]
65-69 years	1225005.22[1712658.73-767572.01]	929.21[1299.11-582.23]	1929071.98[2732258.54-1200247.51]	1339.55[1897.28-833.45]
70-74 years	908218.95[1289665.47-608953.30]	942.22[1337.95-631.75]	1455575.67[2094738.51-963262.93]	1329.93[1913.92-880.11]
75-79 years	566090.77[839703.61-375603.00]	946.85[1404.5-628.24]	903211.25[1340784.21-603790.39]	1252.76[1859.68-837.46]
80-84 years	325556.74[473670.37-212272.47]	888.24[1292.35-579.16]	555349.33[803360.97-360357.41]	1090.39[1577.35-707.54]
85-89 years	131020.89[183972.04-85302.68]	759.42[1066.34-494.43]	235560.50[331323.73-157243.61]	827.42[1163.8-552.33]
90-94 years	31987.99[46966.52-22450.59]	548.82[805.81-385.19]	70775.86[101681.36-49955.6]	586.82[843.07-414.20]
95+ years	6362.60[9366.16-3901.73]	420.80[619.44-258.04]	21883.49[32273.67-13826.83]	555.66[819.49-351.09]
**DALYs**				
<5 years	27588.15[39016.65-18204.73]	8.11[11.48-5.35]	26251.28[37565.23-17571.97]	8.25[11.81-5.52]
5-9 years	27878.23[45265.70-16718.17]	7.86[12.77-4.72]	34490.71[55415.95-21623.09]	10.37[16.66-6.50]
10-14 years	24246.93[38366.40-14548.11]	7.05[11.16-4.23]	34332.20[56599.40-19930.03]	10.63[17.53-6.17]
15-19 years	35420.40[50056.55-23436.05]	11.06[15.63-7.32]	43707.46[73339.50-27302.33]	14.39[24.15-8.99]
20-24 years	46403.26[66562.21-29847.42]	15.29[21.94-9.84]	72546.22[106143.28-49873.85]	24.70[36.13-16.98]
25-29 years	61251.95[86589.67-39278.03]	20.60[29.12-13.21]	67461.20[107605.74-39497.52]	23.18[36.98-13.57]
30-34 years	88760.93[123275.53-60871.81]	29.05[40.35-19.92]	77784.17[123174.55-48239.10]	26.02[41.21-16.14]

35-39 years	90398.04[129762.59-63141.70]	31.94[45.84-22.31]	86545.25[133301.96-53122.15]	31.15[47.98-19.12]
40-44 years	97762.74[137517.70-66895.02]	38.77[54.54-26.53]	89936.35[146069.25-52917.58]	36.25[58.88-21.33]
45-49 years	117050.02[165823.46-78696.26]	49.21[69.71-33.08]	112756.00[188367.64-67067.21]	47.85[79.94-28.46]
50-54 years	132689.54[192578.88-91480.51]	59.78[86.75-41.21]	137028.38[228802.67-83254.14]	61.46[102.63-37.34]
55-59 years	134118.03[189428.88-95546.01]	68.88[97.28-49.07]	148228.73[232223.64-92002.16]	73.74[115.53-45.77]
60-64 years	102956.36[145361.49-76203.91]	66.19[93.46-48.99]	124184.09[191695.71-81266.36]	75.49[116.52-49.40]
65-69 years	106571.91[141083.41-78852.47]	80.84[107.02-59.81]	128878.22[181249.35-90116.28]	89.49[125.86-62.58]
70-74 years	87274.17[115375.65-66428.70]	90.54[119.69-68.92]	105357.26[144586.25-75995.41]	96.26[132.11-69.44]
75-79 years	65982.45[83311.58-52553.61]	110.36[139.35-87.90]	75536.24[101555.07-57558.46]	104.77[140.86-79.83]
80-84 years	42836.98[53779.29-33392.65]	116.88[146.73-91.11]	58950.35[75476.32-46094.37]	115.75[148.19-90.50]
85-89 years	28701.35[35486.63-22898.85]	166.36[205.69-132.73]	34101.97[41672.01-26882.23]	119.79[146.38-94.43]
90-94 years	9999.33[12277.24-7878.34]	171.56[210.64-135.17]	17336.46[20725.79-13599.51]	143.74[171.84-112.76]
95+ years	2272.58[2755.64-1749.23]	150.30[182.25-115.69]	7051.60[8810.35-5273.27]	179.05[223.71-133.90]
**Death**				
<5 years	133.72[218.16-61.30]	0.04[0.06-0.02]	114.82[188.05-70.21]	0.04[0.06-0.02]
5-9 years	88.72[137.01-46.98]	0.03[0.04-0.01]	108.25[144.75-75.69]	0.03[0.04-0.02]
10-14 years	74.87[115.24-38.62]	0.02[0.03-0.01]	68.71[83.37-55.09]	0.02[0.03-0.02]
15-19 years	237.89[332.90-148.94]	0.07[0.1-0.05]	119.94[153.84-92.91]	0.04[0.05-0.03]
20-24 years	342.54[522.16-185.03]	0.11[0.17-0.06]	412.53[518.46-315.82]	0.14[0.18-0.11]
25-29 years	472.84[703.51-290.09]	0.16[0.24-0.10]	235.1[302.65-191.78]	0.08[0.10-0.07]
30-34 years	827.75[1155.06-545.95]	0.27[0.38-0.18]	306.3[371.48-256.06]	0.1[0.12-0.09]
35-39 years	828.96[1153.52-538.3]	0.29[0.41-0.19]	362.82[447.06-303.64]	0.13[0.16-0.11]
40-44 years	923.69[1232.22-662.60]	0.37[0.49-0.26]	277.90[344.85-226.39]	0.11[0.14-0.09]
45-49 years	1223.02[1687.03-834.58]	0.51[0.71-0.35]	469.69[580.05-386.40]	0.20[0.25-0.16]
50-54 years	1548.18[2091.97-1046.67]	0.70[0.94-0.47]	707.67[876.17-580.78]	0.32[0.39-0.26]
55-59 years	1778.80[2297.82-1227.43]	0.91[1.18-0.63]	930.83[1164.33-764.10]	0.46[0.58-0.38]
60-64 years	1612.13[1993.96-1230.42]	1.04[1.28-0.79]	1120.77[1353.08-933.78]	0.68[0.82-0.57]

65-69 years	2352.93[3002.65-1729.72]	1.78[2.28-1.31]	1867.26[2228.88-1547.4]	1.3[1.55-1.07]
70-74 years	2539.78[3197.66-1972.56]	2.63[3.32-2.05]	2154.99[2561.79-1829.21]	1.97[2.34-1.67]
75-79 years	2698.88[3334.4-2046.69]	4.51[5.58-3.42]	2307.14[2790.31-1893.36]	3.20[3.87-2.63]
80-84 years	2371.81[2962.51-1731.97]	6.47[8.08-4.73]	2830.23[3426.12-2317.14]	5.56[6.73-4.55]
85-89 years	2336.38[2872.07-1749.42]	13.54[16.65-10.14]	2427.96[2954.07-1844.27]	8.53[10.38-6.48]
90-94 years	981.14[1231.87-729.92]	16.83[21.14-12.52]	1623.03[1975-1217.60]	13.46[16.38-10.10]
